# Carbonic Anhydrase IX Expression and Treatment Response Measured in Rectal Adenocarcinoma Following Neoadjuvant Chemo-Radiotherapy

**DOI:** 10.3390/ijms24032581

**Published:** 2023-01-30

**Authors:** Emese Sarolta Bádon, Lívia Beke, Attila Mokánszki, Csilla András, Gábor Méhes

**Affiliations:** 1 Department of Pathology, Faculty of Medicine, University of Debrecen, H-4032 Debrecen, Hungary; 2 Department of Oncology, Faculty of Medicine, University of Debrecen, H-4032 Debrecen, Hungary

**Keywords:** rectal adenocarcinoma, neoadjuvant treatment, CAIX expression, *KRAS* status

## Abstract

The overexpression of the pH regulator carbonic anhydrase IX (CAIX) due to hypoxic/metabolic stress was reported in various tumors as an adverse prognostic feature. Our retrospective study aimed to investigate the general pattern and dynamics of CAIX expression in rectal adenocarcinoma following preoperative neoadjuvant therapy (NAT) in matched initial biopsy and surgical resection samples. A total of 40/55 (72.72%) of the post-treatment samples showed partial CAIX expression, frequently in the proximity of hypoxic tumor areas. CAIX expression showed a significant increase in post-treatment tumors (mean% 21.8 ± 24.9 SD vs. 39.4 ± 29.4 SD, *p* < 0.0001), that was not obvious in untreated tumors (mean% 15.0 ± 21.3 SD vs. 20 ± 23.02, *p* = 0.073). CAIXhigh phenotype was associated with mutant *KRAS* status and lack of pathological regression (WHO Tumor Regression Grade 4 and 5). However, the adverse effect of CAIX on overall or progression-free survival could not be statistically confirmed. In conclusion, the dynamic upregulation of CAIX expression is a general feature of rectal adenocarcinoma following neoadjuvant chemo-radiotherapy indicating therapy-induced metabolic reprogramming and cellular adaptation. A synergism of the CAIX-associated regulatory pathways and the mutant *KRAS* oncogenic signaling most likely contributes to therapy resistance and survival of residual cancer.

## 1. Introduction

The treatment efficacy in colorectal carcinoma (CRC) depends on standard variables, such as the location, the histology subtype, the predictive genetic background, and the stage of the tumor [1]. However, individual biological features determine functional differences resulting in heterogeneous responses to chemo- and radiotherapy. Inadequate perfusion and related tissue hypoxia belong to the yet-underscored causes of regional tumor resistance [2,3]. Hypoxia interferes with basic cellular and physiological processes, including cell proliferation, quiescence and apoptosis, glucose metabolism, pH regulation, and angiogenesis, contributing to the survival of cancer cells [4,5]. The activation of adaptive mechanisms circumventing hypoxic damage is a hallmark of aggressive cancers with poor prognoses. As part of the adaptive process, intracellular acidosis in cancer cells with anaerobic metabolism is compensated at the expense of the extracellular pH, inducing functional changes in the hypoxic microenvironment, further promoting resistance and cancer progression [2,4,6].

Carbonic anhydrases belong to a family of zinc metalloenzyme proteins that catalyze the rapid and reversible hydration of carbonic dioxide to bicarbonate and protons as part of the cellular pH regulatory system. Carbonic anhydrase IX (CAIX) plays a significant role in the adaptive response to hypoxia concerted by hypoxia-inducible factor-1 (HIF-1), both in normal and cancer cells. Moreover, by the contribution to microenvironmental acidosis, CAIX is involved in tumor-stroma and tumor-immune cell interactions and accelerates extracellular matrix degradation, thereby facilitating the invasion and proliferation of acid-resistant cells [7,8,9,10,11]. The overexpression of CAIX due to hypoxic stress was reported as an adverse prognostic feature in various tumors [12,13,14,15,16,17,18,19]. CAIX-related changes are considered complex mechanisms that classical anticancer drugs and biological therapies cannot effectively exploit [20,21]. The inhibition of the pH regulator CAIX to increase cellular vulnerability and restore acidic extracellular pH resulted in impaired tumor growth and reduced metastatic potential of various types of tumor cells [8,20].

Earlier data suggest a prognostic role of CAIX upregulation due to hypoxia in colorectal carcinoma [17,22,23]. However, its distribution and dynamic nature have not been studied in detail. According to our hypothesis, anti-tumor therapies, such as neoadjuvant therapy have a basic effect on cancer cell metabolism and perfusion which is also reflected by cellular adaptation, such as measurable changes in CAIX expression. As a model system, rectal adenocarcinoma samples taken before (diagnostic biopsy) and after (surgical resection) were evaluated and compared with untreated cases for CAIX expression and other available variables of the disease. Our retrospective study aimed to investigate (i) the general pattern of CAIX expression in rectal adenocarcinoma as demonstrated by immunohistochemistry; (ii) the expression of CAIX in rectal adenocarcinomas that were untreated (UT) and following preoperative neoadjuvant therapy (NAT); (iii) the relationship of CAIX expression in pretreatment biopsy samples compared to treated surgical specimens (control group); (iv) the correlation of CAIX expression with pathological and biological status including the cell proliferation, tumor regression grade, and the *KRAS* mutational status; (v) the effect of CAIX on patient survival.

## 2. Results

### 2.1. CAIX Expression Pattern in Rectal Adenocarcinoma Samples

**Figure 1:**
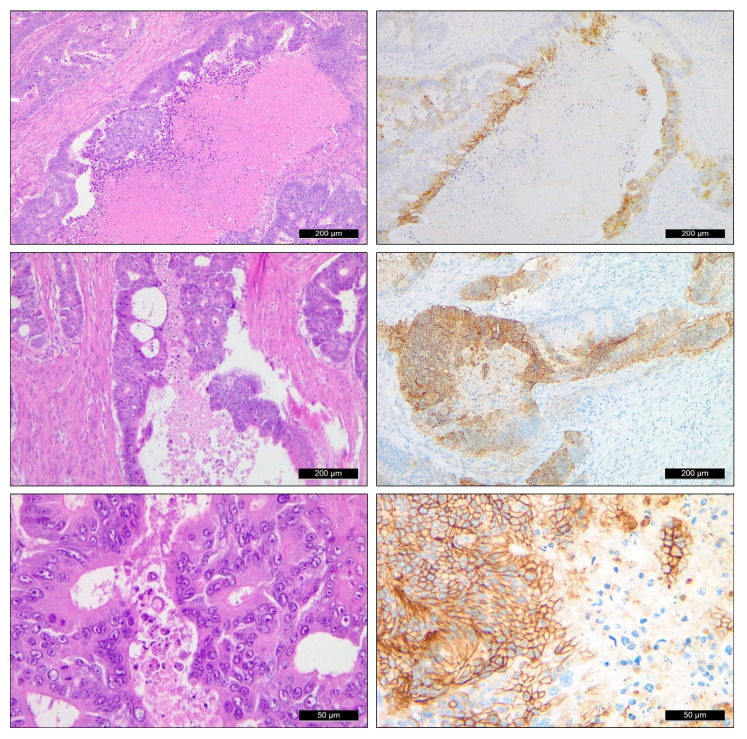
CAIX expression pattern in rectal adenocarcinoma. Conventional HE staining (**left**, 10×) and CAIX immunostaining (**right**) of equivalent tumor areas presenting selective cell membrane staining. CAIX expression was predominantly found in association with necrotic tumor areas with variable heterogeneity (**top** and **middle** inserts, 10×). Robust membrane labeling in a uniform fashion was seen in some solid areas of the tumor (**bottom row**, 40×).

CAIX expression occurred in a highly variable form and amount in the evaluated rectal adenocarcinoma samples. In general, selective staining of tumor cells was seen, while normal/unaffected rectal mucosa proved to be negative for CAIX. Within tumor areas, characteristic and selective cell membrane staining was observed with variable intensity. Regarding the distribution in individual cases, a strong association with necrotic foci could be recognized, presenting a strong perinecrotic tumor cell labeling and a dynamic loss toward the more distant layers. The relation to necrosis was closer evaluated in surgical resection samples: NAT resections presented with necrosis in 40 out of the 55 evaluated samples (72.72%). CAIX positivity was seen in 24 (60%) around the perinecrotic area and no CAIX expression was identified in 16 (40%) cases. In the 34 cases of UT resected samples, 29 out of the 34 evaluated samples (85.29%) were identified with necrotic areas, of which 26 (89.65%) showed CAIX positivity around the necrotic area, while 3 (10.34%) samples remained negative for CAIX (Figure 1).

**Figure 2:**
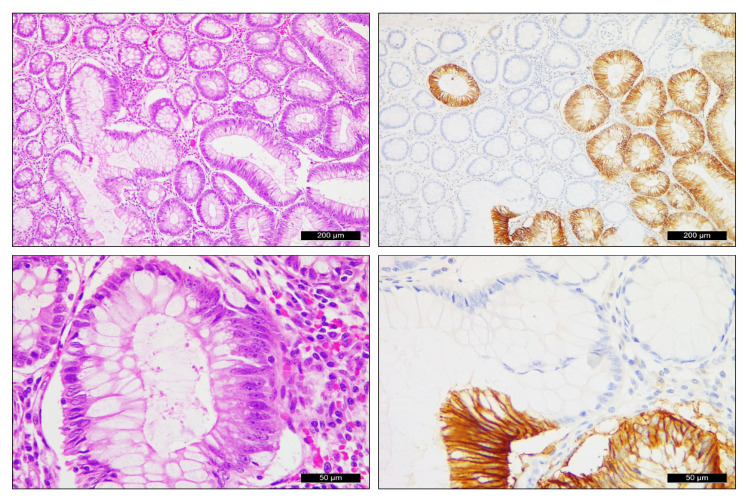
Selective CAIX expression in areas with moderate to severe dysplasia next to rectal adenocarcinoma (**upper row** 10×, **lower row** 40×).

Interestingly, CAIX expression in areas with severe dysplasia could also be frequently identified, well separating the area from the normal epithelium or low-grade changes. Moderate to severe dysplasia with characteristic membrane CAIX expression was identified in 5/55 (9.09%) of the NAT surgical specimens and 5/34 (14.7%) of the UT surgical specimens (Figure 2).

**Figure 3:**
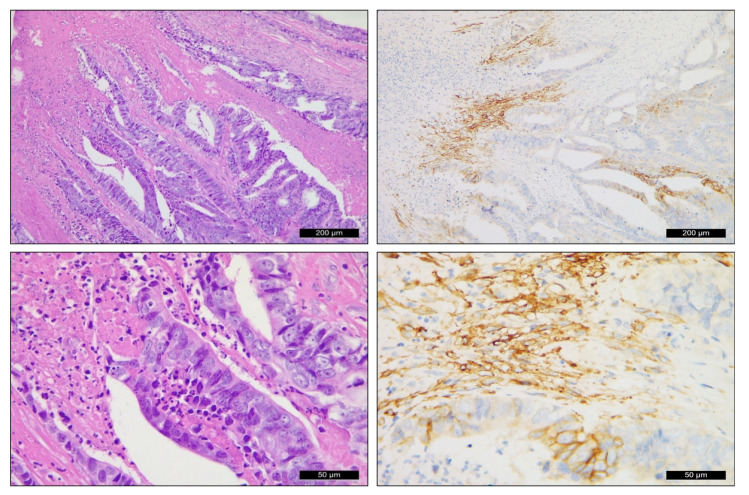
CAIX expression in epithelial and mesenchymal cells of the neostroma in rectal carcinoma (**upper row** 10×, **lower row** 40×).

Further to the neoplastic cell clusters of glandular epithelial origin, CAIX expression within the tumor neostroma was also seen. Increased CAIX expression within the tumor stroma was observed in 24/55 (43.63%) of the NAT surgical samples while 31/55 (57.37%) were stroma CAIX-negative. In the UT group of 34 cases, only 9/34 (26.47%) of the surgical specimens showed stromal positivity for CAIX (Figure 3).

### 2.2. Expression Dynamics of CAIX in Neoadjuvant-Treated Rectal (NAT) Adenocarcinomas (n = 55)

**Figure 4:**
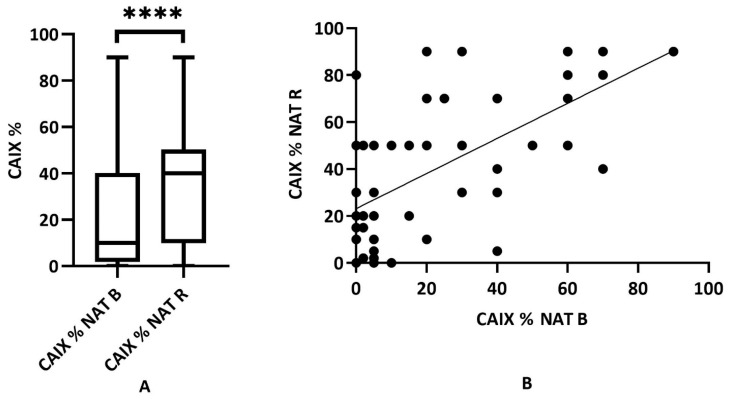
Distribution of CAIX expression in NAT biopsies (NAT B) and NAT surgical resection samples (NAT R) (*n* = 55). (**A**) Cumulative result of CAIX tumor labeling (range 0–90% for both groups, mean 21.8 ± 24.9 SD vs. 39.4 ± 29.4 SD, respectively), where the CAIX labeling is significantly different (*p* < 0.0001, ****: statistically highly significant); (**B**) correlation of CAIX expression determined between NAT B (pretreatment) and NAT R (post-treatment) samples of the NAT cancer group (*p* < 0.0001, rho: 0.5654).

CAIX expression was quantified in all samples by defining the proportion of positive labeling in the percentage of the tumor area. The labeling in the individual samples from before and after treatment was compared. In the statistical analysis of the NAT biopsy and the NAT surgical specimen, we found a marked increase in CAIX following the treatment (mean 21.8 ± 24.9 SD vs. 39.4 ± 29.4 SD) which was found to be statistically significant (Wilcoxon matched rank test *p* < 0.0001). Further, a positive mathematical correlation between the biopsy and the surgical sample was demonstrated (Spearman correlation test *p* < 0.0001, rho: 0.5654) (Figure 4A,B).

For further comparison, the 55 NAT and 34 UT rectal carcinomas were split exactly by the median CAIX percentage and classified as CAIXlow and CAIXhigh carcinomas. Available clinicopathological data were evaluated to represent potential differences associated with CAIX status. Most importantly, statistical significance between CAIX expression and mutant *KRAS* status could be established (biopsy *p* < 0.0151; surgical specimens *p* < 0.0316), while no correlation with any other clinicopathological parameters was found. The results are shown in Table 1.

### 2.3. Immunohistochemical CAIX Expression of Untreated (UT) Rectal Adenocarcinoma (n = 34)

**Figure 5:**
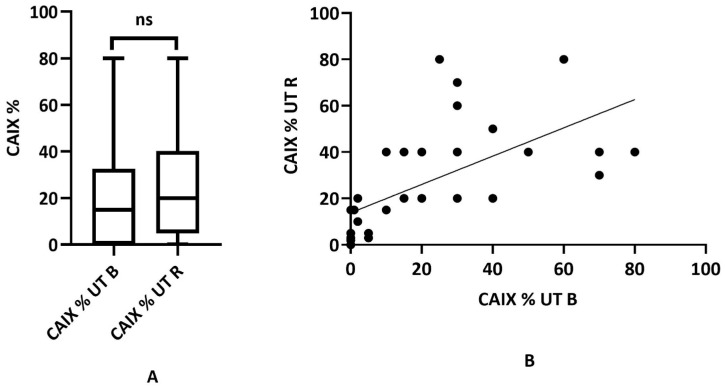
Distribution of CAIX expression in untreated biopsies (UT B) and matching surgical resection samples (UT R) (*n* = 34). (**A**) The range of CAIX tumor labeling was 0–80% for both sample types, but a significant difference is not provided (mean 15.0 ± 21.3 SD vs. 20 ± 23.02, *p* = 0.073, ns: statistically not significant); (**B**) correlation between UT B and UT R CAIX expression in the UT cancer group (*p* < 0.0001, rho: 0.8077).

Untreated rectal adenocarcinoma samples were evaluated the same way as previously presented. CAIX expression was highly variable with values ranging from 0 to 80%. In contrast to the NAT tumor group, the statistical analysis of the UT biopsy and the UT surgical specimen did not result in statistical difference regarding CAIX expression (mean 15.0 ± 21.3 SD vs. 20.0 ± 23.02, Wilcoxon matched rank test *p* < 0.073) but the correlation between the biopsy and surgical samples could be well established (Spearman correlation test *p* < 0.0001, rho: 0.8077) (Figure 5A,B).

Classification as CAIXlow and CAIXhigh based on the median CAIX score in UT samples was followed by the analysis of related clinicopathological data. Similar to the NAT group of carcinomas, we found a statistically significant correlation between CAIX expression and the *KRAS* status when biopsy CAIX values were considered (biopsy *p* < 0.0454; surgical specimens *p* < 0.0921). All other evaluated parameters were independent of the CAIX status (Table 2).

### 2.4. Tumor Regression Grade and CAIX in NAT Biopsy and NAT Surgical Specimens (n = 55)

**Figure 6:**
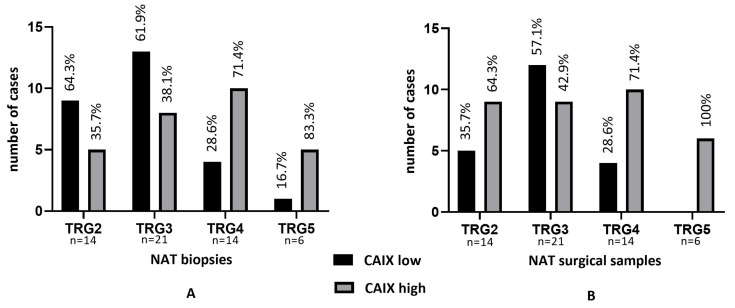
Tumor regression grade (TRG) as defined in post-treatment NAT surgical resection samples and CAIX expression in pre-treatment NAT biopsy (**A**) and post-treatment NAT surgical samples (**B**). Response failure (TRG4–5) is predominantly associated with the CAIXhigh phenotype.

To investigate the effect of neoadjuvant treatment, we determined the tumor regression grade (TRG) according to the WHO recommendations in the NAT surgical specimens that were obtained following therapy. The resulting TRG was correlated with CAIX expression of the same post-treatment sample, but also with the pretreatment biopsy scores for potential predictive features. When evaluating pretreatment biopsies, the majority of TRG2–3 cases were associated with CAIXlow (64.3 and 61.9%), and reverse, TRG4–5 cases with the CAIXhigh phenotype (71.4 and 83.3%), indicating an increased potential of treatment failure in initially CAIXhigh tumors. In contrast, CAIX expression proved to be generally increased following NAT, also contributing to elevated scores in residual tumors with low regression grades. Nevertheless, tumors with limited/no treatment response (TRG4–5) presented with the CAIXhigh phenotype (71.4% and 100.0%) (Figure 6A,B).

### 2.5. KRAS Status and CAIX Expression in NAT and UT

**Figure 7:**
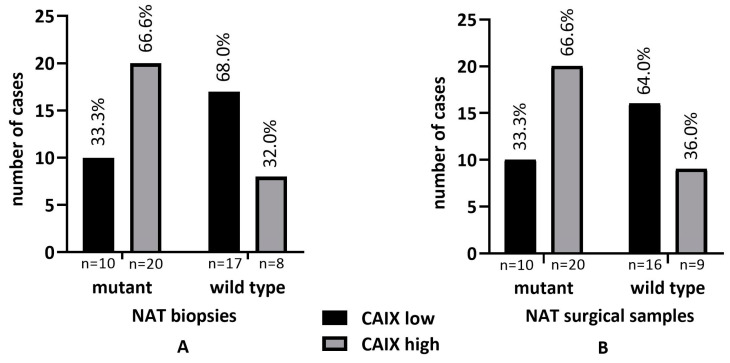

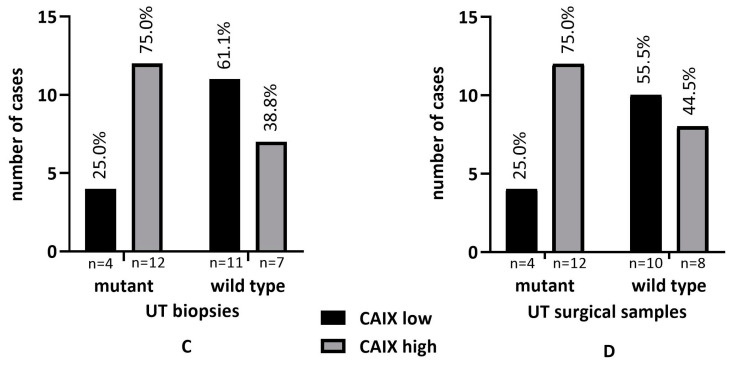
Correlation between *KRAS* status and CAIX expression in biopsies and post-treatment surgical samples of NAT (**A**,**B**) and biopsies and untreated surgical samples of UT (*n* = 34) rectal adenocarcinoma cases (**C**,**D**).

We determined the *KRAS* mutational profile in all rectal adenocarcinoma samples that were included which allowed a comparison with CAIX expression data. The comparison of *KRAS* mutant and wild-type tumor groups confirmed significant differences as *KRAS* mutant samples presented with much higher CAIX scores and the correlation of CAIX expression proved to be statistically significant in NAT biopsies, surgical samples, and UT biopsies, but not in UT surgical samples, according to the Fisher’s exact test (*p* < 0.05) (Figure 7A–D).

**Figure 8:**
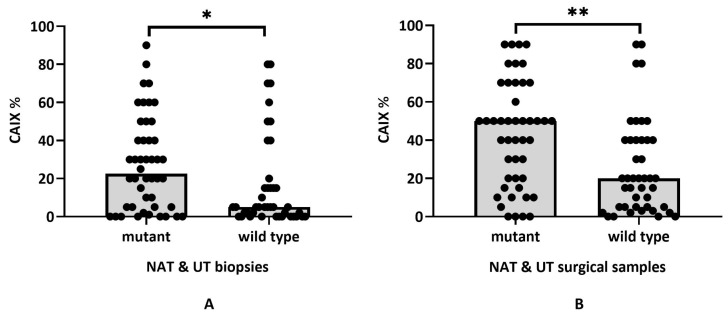
*KRAS* status and CAIX expression in combined NAT and UT rectal adenocarcinoma samples (*n* = 89). Distribution of CAIX scores in *KRAS* mutant and wild-type tumors in biopsies (**A**) and surgical resection samples (**B**) Mutant *KRAS* tumors present with significantly higher overall CAIX scores (* *p* = 0.0138; ** *p* = 0.002).

Since the *KRAS* mutant rectal adenocarcinomas were significantly more represented in the CAIXhigh group uniformly for both NAT and UT samples, we also performed a combined analysis using the exact CAIX scores related to the *KRAS* status in the unified rectal carcinoma cohort (*n* = 89, mutant *n* = 46, and wild type *n* = 43). As expected, the CAIX expression score proved to be significantly higher in *KRAS* mutant cases (initial biopsies: wild-type mean 16.28 ± 24.34 vs. mutant mean 27.67 ± 24.60; Mann–Whitney test *p* = 0.0138; NAT & UT surgical samples: wild-type mean 25.51 ± 25.10 vs. 43.15 ± 27.62; Mann–Whitney test *p* = 0.002) (Figure 8A,B).

### 2.6. Patient Survival and CAIX Expression in NAT and UT Samples

Next, we were looking at the correlation of CAIX expression in NAT biopsy and surgery samples with overall survival (OS) and progression-free survival (PFS). According to the statistical analysis (Kaplan–Meier curves), no significant difference in OS or PFS could be measured between the CAIXhigh and CAIXlow categories (OS biopsies *p* = 0.9769 vs. surgical samples *p* = 0.6585; PFS biopsies *p* = 0.2129 vs. 0.7382).

Similarly, the evaluation of UT biopsy and surgery samples did not result in a significant correlation between OS and PFS and the CAIXhigh and CAIXlow expression groups (OS biopsies *p* = 0.1620 vs. surgical samples *p* = 0.7940; PFS biopsies *p* = 0.4830 vs. *p* = 0.1380).

## 3. Materials and Methods

### 3.1. Patients and Study Design

The study was based on archived formaldehyde fixed and paraffin-embedded histological samples diagnosed and stored at the Department of Pathology, Clinical Center, University of Debrecen. The investigation was performed in agreement with the highest ethical standards and covered by the national ethical approval (IRB reference number: 60355-2/2016/EKU and IV/8465-3/2021/EKU).

We examined 55 matched initial biopsies and post-treatment surgical samples that were obtained from patients with rectal adenocarcinoma diagnosis undergoing preoperative neoadjuvant chemo-radiotherapy (NAT group). In addition, 34 matched biopsies and surgical samples were included from untreated rectal adenocarcinoma patients (UT group). Patients undergoing NAT received the following treatments based on the prescribed protocols: Capecitabine monotherapy protocol 2500 mg/m^2^; Mayo protocol: Fluorouracil (5-FU) 425 mg/m^2^ and calcium folinate (FOL) 20 mg/m^2^ and Fluorouracil (5-FU) monotherapy protocol 500 mg/m^2^. All patients received radiotherapy (total dose of 50.4 *Gy* (*1.8 Gy*/day, 5 days/week) together with chemotherapy, two cases received only radiotherapy.

The selection criteria included that the basic condition of the treatment was at least TNM Stage II, and both the biopsy and the resection sample contained representative tumor tissue for accurate analysis of the CAIX expression. Basic clinical and histopathological parameters, including sex, tumor grade and depth, presence/absence of metastasis, mucinous phenotype, *KRAS* status, type of neoadjuvant treatment, and tumor regression grade, were compared. The characteristics of the study group are summarized in Table 3.

### 3.2. Immunohistochemical Detection of CAIX

Tumor samples were received after primary colonoscopic biopsy and surgical tumor resection in PBS-buffered formaldehyde solution (4%) for standard tissue processing. FFPE embedding and histology were performed in the Department of Pathology, University of Debrecen. Sections that were 3 µm were cut on silanized slides from the selected blocks and immunohistochemical staining was performed as usual.

The IHC procedure was as follows: Carbonic Anhydrase IX/CAIX (clone EP161) rabbit monoclonal antibody (cat. nr. 379R-16, Cell Marque/Sigma-Aldrich, Rocklin, CA, USA), dilution was 1/200. For antigen retrieval, the Cell Conditioning Solution (ULTRA CC1) Tris-based buffer (pH 8.5, cat. nr. 950-224) was used for 48 min at 100 °C. The incubation was for 48 min at 37 °C in a BenchMark Ultra immunostaining machine (Roche Diagnostics, Tucson, AZ, USA). The reaction was detected with the OptiView DAB IHC Detection kit (cat. no. 760–700), followed by Hematoxylin II (cat. nr. 790–2208) staining according to the manufacturer’s instructions.

The immunostainings were evaluated using light microscopy independently by two histopathologists (EB, GM) in a blinded fashion. If conflicting values were obtained, the decision was made by mutual agreement following personal discussion. The expression of CAIX was quantified using a visual grading system based on the extent of staining (percentage of positive tumor cells: 0–100%). A median value was calculated from the obtained percentage expression values. Depending on the median value, the CAIXlow and CAIXhigh groups were formed for further analysis: the CAIXlow group included all cases with CAIX values below the median, including negative staining results and the CAIXhigh group represented cases with values equal to/above the median. CAIXlow and CAIXhigh categories were separately evaluated within the NAT and UT groups. For comparison, we investigated the distribution of CAIX and general features of CAIXlow and CAIXhigh categories in both initial biopsy and surgical resection samples.

### 3.3. Tumor DNA Extraction from FFPE Tissue Samples

Tumor samples with a >20% tumor percentage were selected based on H & E staining for molecular analysis. Genomic DNA was extracted from FFPE tissues using QIAamp DNA FFPE Tissue Kit (Qiagen, Hilden, Germany). The DNA concentration was measured in the Qubit dsDNA HS Assay Kit using a Qubit 4.0 Fluorometer (Thermo Fisher Scientific, Waltham, MA, USA).

### 3.4. Mutation Testing Using StripAssay

Reverse hybridization was carried out using the *KRAS* XLStripAssay according to the manufacturer’s protocol (ViennaLab Diagnostics, Vienna, Austria). The assay certified for human in vitro diagnostics (IVD) covers 29 clinically relevant mutations of the *KRAS* gene. Hybridization strips were aligned using the standardized layout supplied with the reagents for interpretation. Positive bands allowed the determination of the *KRAS* mutant status and the accurate identification of individual *KRAS* variants.

### 3.5. Statistical Analysis

We used GraphPad Prism 8 statistical software (Dotmatics, Boston, MA, USA) from which Wilcoxon matched rank test, Spearman correlation test, Mann–Whithney U test, and the VassarStats (http://vassarstats.net, accessed on 4 January 2023) online software was used to apply the Fisher’s exact test which was to evaluate the statistically significant association between the expression of proteins and clinical and histopathological parameters. Only *p* < 0.05 was considered significant.

## 4. Discussion

The present study focuses on the dynamic expression of hypoxic stress-related CAIX in rectal adenocarcinoma determined by immunohistochemistry. Diagnostic rectoscope samples and post-treatment surgical resection samples from neoadjuvant-treated cases and untreated control cases were compared. As expected, CAIX expression was limited to a variable fraction of cancer cells in a highly specific cell membrane localization. The extent of CAIX was heterogenous and individual, stretching over a wide spectrum from 0 to 90% of the tumor cell compartment. CAIX characteristically presented in the proximity of tumor necrotic foci, but larger solid areas lacking necrosis were also seen. Moreover, areas with severe dysplasia could be distinct by increased CAIX labeling compared to the surrounding normal glandular epithelium. Due to the intratumoral complexity of the labeling, the sample cohort was split by creating the CAIXlow and CAIXhigh categories for further analysis. In our study, the CAIX expression showed no statistical correlation with most of the conventional clinic-pathological parameters. Similar results were published by Korkeila et al., who investigated CAIX expression in 166 samples of rectal carcinoma and found that the CAIX expression pattern was independent of the selected parameters, e.g., size, nodal status, or grade of the tumor [22]. Kovacova and Hodorova also concluded that CAIX expression was not significantly associated with sex, grade of tumor, nodal status, or presence/absence of metastasis [23]. Tupa et al. also did not detect a significant difference between CAIX expression and clinical-morphological characteristics [24].

In our current analysis, we aimed to compare CAIX expression dynamics by including pretreatment, post-treatment, and untreated control rectal carcinoma samples. We hypothesized that chemo-radiotherapy-related stress and perfusion deficiency are associated with hypoxic damage and the induction of adaptive mechanisms. As an important observation, a significant increase of CAIX could be demonstrated following chemo-radiotherapy in the surgical resection samples (NAT), while this was not obvious in the untreated (UT) tumor samples. In a single report, Guedj et al. in their study pointed out that CAIX expression was significantly lower in pretreatment biopsy specimens from responders than in non-responders [25]. In our samples, the local effects of chemo-radiotherapy were visible by standard histology, including tissue remodeling following mass tissue damage and tumor necrosis. Residual tumor areas persisted with reduced cell proliferation activity (determined by the Ki-67 labeling index), a regressive feature that is related to the direct cytotoxic effect and loss of proper tissue perfusion. In line with other histological changes, the overexpression of CAIX demonstrated here may indicate hypoxia-driven adaptation which potentially contributes to the resistance mechanisms leading to limited treatment response rates.

Aggressive tumor behavior and treatment resistance are associated with the occurrence of driver mutations. In this cohort, we were able to evaluate the relationship of CAIX expression with the *KRAS* mutational status which proved to be correlated, as *KRAS* mutant rectal adenocarcinomas presented initially and following neoadjuvant therapy with significantly higher CAIX scores and CAIXhigh phenotype. This relation was observed independently of the treatment status and indicates a close interaction of mutant *KRAS*-activated MAPK pathway with cancer cell metabolism and oxygen demand in rectal carcinoma. This mechanism is supported by McDonald et al., who examined the pH regulation by CAIX in pancreatic ductal adenocarcinoma cells with activated *KRAS*. In response to hypoxia, *KRAS*-activated pancreatic ductal adenocarcinoma cells presented with CAIX overexpression through the stabilization of HIF1A and HIF2A, as an adaptive process to maintain pH and glycolysis. This study suggests CAIX functions as a critical vulnerability in *KRAS*-driven pancreatic ductal adenocarcinoma [15].

A widely used measure of therapy failure is the tumor regression grade which can be estimated and classified in post-treatment surgical samples [26,27]. The analysis of CAIX expression presented that the lack of significant tumor regression (TRG 4–5) was associated with the CAIXhigh phenotype determined in both pre-treatment biopsies and post-treatment rectal carcinoma specimens. This indicates a potentially increased resistance of CAIX-expressing cancer cells and suggests the utility of CAIX scoring for the characterization of post-treatment tumors [28,29,30].

Despite these particular correlations, the true prognostic role of CAIX remains rather controversial. Our study was not able to demonstrate long-term survival differences between CAIXlow and CAIXhigh disease groups. However, earlier findings are contradictory regarding the predictive significance of CAIX, which might be primarily associated with the complexity of target determination and sample cohorts. CAIX expression was reported to be significantly associated with decreased disease-specific survival [2]. In reverse, rectal cancer patients with negative or weak CAIX staining intensity had significantly longer disease-free survival [22]. Kuijik et al., in their review study stated that patients with high CAIX expression in colorectal carcinomas could expect shorter disease-free and progression-free survival, and worse metastasis-free survival [17]. On the contrary, the analysis of 539 colorectal patients for cancer-associated carbonic anhydrases did not result in a significant correlation between major clinical parameters, survival, and extent of CAIX immunostaining [31]. Debucquoy et al. also claimed, based on their results, that CAIX has no prognostic significance [28]. As an interesting finding, CAIX mean protein expression intensity was significantly upregulated in ulcerative colitis-associated colorectal carcinoma compared with sporadic colorectal carcinoma suggesting CAIX as a marker to address cancer etiology [29]. The discrepancies may be explained by the variability of the population composition, histological subtypes, antibodies, detection systems, statistical methods, and lack of standardization.

It is getting increasingly clear that individual sensitivity to hypoxia and acidosis is a critical feature of cancer. Despite many debatable points, our present work seems to extend the current knowledge with several important findings: (i) CAIX is inducible and significantly increased by the neoadjuvant chemo-radiotherapy in rectal adenocarcinoma; (ii) failure in tumor response defined by TRG following neoadjuvant therapy is associated with high CAIX expression; (iii) the mutant *KRAS* status is associated with increased CAIX expression and their synergistic effects can be suspected in cancer progression and treatment failure. Studies on CAIX expression in rectal adenocarcinoma allowed a deeper insight into the phenotypic changes of the cancer tissue upon hypoxic stress but the clinical significance of the hypoxia-CAIX axis in individual cases is still to be established.

## Figures and Tables

**Table 1:** Statistical distribution of CAIX expression concerning clinicopathological parameters of NAT rectal adenocarcinoma.

Clinicopathological Parameters in Treated Patients	Quantity of CAIX Expression in Biopsy		Quantity of CAIX Expression in Resection		Fisher’s Exact Test in Biopsy	Fisher’s Exact Test in Resection
	*CAIXlow*	*CAIXhigh*	*CAIXlow*	*CAIXhigh*		
**Total (*n* = 55)**	27	28	26	29	*p* > 0.9999	
**Sex**						*p* > 0.7807
Male (*n* = 35)	19	16	18	17	*p* > 0.4032	
Female (*n* = 20)	8	12	9	11		
**Grade of tumor**						
G2 (*n* = 36)	20	16	19	17		
G3 (*n* = 17)	6	11	6	11	*p* > 0.4466	*p* > 0.5609
G4 (*n* = 2)	1	1	1	1		
**Lymph nodes**						
positive (*n* = 16)	-	-	9	7	-	*p* > 0.5532
negative (*n* = 39)	-	-	17	22		
**Metastasis**						
present (*n* = 11)	-	-	6	5	-	*p* > 0.9999
absent (*n* = 44)	-	-	24	20		
**Necrosis**						
present (*n*(B) = 2; *n*(R) = 24)	8	7	9	15	*p* > 0.4706	*p* > 0.1965
absent (*n*(B) = 15; *n*(R) = 16)	0	2	10	6		
**Stroma**						
present (*n*(B) = 4; *n*(R) = 24)	2	2	13	11	*p* > 0.9999	*p* > 0.4183
absent (*n*(B) = 51; *n*(R) = 31)	25	26	13	19		
**Tumor-infiltrating lymphocytes**						
present (*n*(R) = 24)	-	-	13	11	-	*p* > 0.7910
absent (*n*(R) = 31)	-	-	18	13		
**Mucinous phenotype**						
present (*n* = 11)	3	8	4	7	*p* > 0.1771	*p* > 0.5104
absent (*n* = 44)	24	20	22	22		
**Tumor regression grade**						
TRG2 (*n* = 14)	9	5	5	9	*p* > 0.0660	*p* > 0.0590
TRG3 (*n* = 21)	13	8	12	9		
TRG4 (*n* = 14)	4	10	4	10		
TRG5 (*n* = 6)	1	5	0	6		
***KRAS* status**						
wild-type (*n* = 25)	17	8	16	9	***p* < 0.0151**	***p* < 0.0316**
mutant (*n* = 30)	10	20	10	20		

**Table 2:** Statistical distribution of CAIX expression concerning clinicopathological parameters of untreated (control) rectal adenocarcinoma samples.

Clinicopathological Parameters in Untreated Patients	Quantity of CAIX Expression in Biopsy		Quantity of CAIX Expression in Resection		Fisher’s Exact Test in Biopsy	Fisher’s Exact Test in Resection
	*CAIXlow*	*CAIXhigh*	*CAIXlow*	*CAIXhigh*		
**Total (*n* = 34)**	15	19	14	20	*p* > 0.9999	
**Sex**						*p* > 0.2714
Male (*n* = 23)	12	11	12	11	*p* > 0.4768	
Female (*n* = 11)	4	7	3	8		
**Grade of tumor**						
G1 (*n*(B) = 1, *n*(R) = 1)	0	1	0	1	*p* > 0.3547	*p* > 0.9068
G2 (*n*(B) = 28, *n*(R) = 19)	14	14	9	10		
G3 (*n*(B) = 5, *n*(R) = 13)	1	4	5	8		
G4 (*n* = 1 R)	-	-	0	1		
**Lymph nodes**						
positive (*n* = 17)	-	-	6	11	-	*p* > 0.7283
negative (*n* = 17)	-	-	8	9		
**Metastasis**						
present (*n* = 5)	-	-	1	4	-	*p* > 0.3786
absent (*n* = 29)	-	-	13	16		
**Necrosis**						
present (*n*(B) = 2; *n*(R) = 26)	0	2	9	17	*p* > 0.4667	*p* > 0.5320
absent (*n*(B) = 4; *n*(R) = 3)	2	2	0	3		
**Stroma**						
present (*n*(B) = 0; *n*(R) = 9)	-	-	3	6	-	*p* > 0.9999
absent (*n*(B) = 34; *n*(R) = 25)	14	20	8	17		
**Tumor-infiltrating lymphocytes**						
present (*n*(R) = 19)	-	-	5	9	-	*p* > 0.4953
absent (*n*(R) = 15)	-	-	10	10		
**Mucinous phenotype**						
present (*n* = 6)	2	4	1	5	*p* > 0.6722	*p* > 0.3636
absent (*n* = 28)	13	15	13	15		
***KRAS* status**						
Wild-type (*n* = 18)	11	7	10	8	***p* < 0.0454**	*p* > 0.0921
mutant (*n* = 16)	4	12	4	12		

**Table 3:** Clinico-pathological and biological characteristics of rectal adenocarcinomas treated with neo-adjuvant therapy (NAT) and untreated controls (UT) evaluated in the study.

	NAT Treated Patients		UT (Control) Patients	
Characteristics	Classification	% (N = 55)	Classification	% (N = 34)
Sex	Male	35	Male	23
	Female	20	Female	11
Age	Male (range)	68.6 (41–85)	Male	68.43 (51–84)
	Female (range)	66.2 (52–84)	Female	69 (52–83)
Histological grade	G1	0	G1	1
	G2	36	G2	19
	G3	17	G3	13
	G4	2	G4	1
Tumor depth	T1	0	T1	3
	T2	29	T2	9
	T3	25	T3	19
	T4	1	T4	3
	Nx	0	Nx	2
	N0	33	N0	10
	N1	15	N1	14
	N2	7	N2	8
	Mx	51	Mx	0
	M0	0	M0	0
	M1 (liver)	4	M1 (liver)	1
Mucinous phenotype	Present	11	Present	6
	Absent	44	Absent	28
*KRAS* status	Wild-type	25	Wild-type	18
	Mutant	30	Mutant	16
Type of neoadjuvant treatment	Capecitabine & RT	32		
	5FU & FOL & RT	16		
	5FU &RT	5		
	RT	2		
Tumor regresssion grade	R1	0		
	R2	14		
	R3	21		
	R4	14		
	R5	6		

## Data Availability

The data presented in this study are available on request from the corresponding author. The data are not publicly available to protect the rights of patients.
